# The effect of piling behavior on the production and mortality of free-range laying hens

**DOI:** 10.1016/j.psj.2023.102989

**Published:** 2023-08-05

**Authors:** David Armstrong, Lucy Asher, Ann Rayner, Halima Ngidda, Bryony Sharma, Helen Gray

**Affiliations:** ⁎ School of Natural and Environmental Sciences, Newcastle University, Newcastle upon Tyne NE1 7RU, United Kingdom; † FAI Farms Ltd., The Barn, Wytham, Oxfordshire OX2 8QJ, United Kingdom

**Keywords:** smothering, chicken, crowding, welfare, egg

## Abstract

Piling, a dense cluster of hens, is a behavior of major concern to the cage-free egg industry. It can cause large numbers of mortalities at which point it is considered smothering. The aim of this study was to quantify whether piling can also have consequences on production and non-smothering mortalities, which have not previously been described. Additionally, we aimed to describe characteristics of piling behavior relevant to for management. Video footage from 12 flocks of laying hens was analyzed for piling behavior across 3 wks. Production and mortality data were accessed using an integrated online management system. Bayesian linear mixed-effect models were used for formal statistical testing of the relationships between piling and production. Accounting for some missing data, a total of 252 d amounting to approximately 15,624 h were analyzed for the presence or absence of piling behavior, which we believe constitutes the largest analysis of piling behavior in the scientific literature to date. All flocks observed exhibited piling behavior even if they had no history of smothering. On average, flocks piled more than 4 times per day for around 44 min per event and the peak piling time occurred between 1300 and 1359. We found that the number of piling events was associated with a reduction in the number of eggs produced the next day; based on the average of 4 piles per day this amounted to 7.35 fewer eggs per 1,000 birds per day. Contrary to our hypothesis, we found total piling duration per day was positively associated with fewer Grade B eggs, with a decrease of 0.74 Grade B eggs for every hour of piling per day. No relationship was observed between piling and mortality not attributed to smothering. We discuss possible causes and explanations for these results including birds’ response to stress, crowd dynamics, and daily rhythms. Here we show the potential for piling behavior to have sublethal consequences on production even in the absence of smothering-related deaths.

## INTRODUCTION

Piling is an undesirable group behavior that is prevalent in the cage-free egg industry. It describes a behavior by which individual hens come together to form a large mass that behaves almost as if it is a single entity (Barrett et al., 2014). Piling can be a precursor to smothering, which is defined as when piling behavior results in the death of one or more of the participating birds (Gray et al., 2020; Herbert et al., 2021). Not all piles are smothers, but all smothers are preceded by piling behavior. Smothering is believed to be common, with 50% of free-range flocks reported to be affected in a UK survey (Barrett et al., 2014) and a further study suggesting it could account for around one sixth of mortalities across the UK industry annually (Nicol, 2015).

Despite the scale of the issue, relatively little is known about smothering. Within the industry much of the knowledge base is anecdotal. What is widely accepted is a stratification of piling into three different categories: nest box, panic, and creeping or recurrent (for the purposes of this paper the term creeping will be preferred) (Bright and Johnson, 2011; Gebhardt-Henrich and Stratmann, 2016; Gray et al., 2020). A nest box event occurs in the laying area of a shed when a large number of birds attempt to access the same nest box at the same time, it often occurs early in the flock cycle when hens are learning about their environment and becomes less of an issue as they age (Bright and Johnson, 2011). There is some evidence that nest box smothers are impacted by nest box design (Rayner et al., 2016) and location (Giersberg et al., 2019). A panic event happens when a stimulus startles the flock causing quick, synchronized movement. A creeping event, however, does not appear to be triggered by a particular stimulus and builds up gradually (Bright and Johnson, 2011; Gray et al., 2020). This paper is primarily interested in creeping piles, which are the most common precursor to smothering (Barrett et al., 2014) and the least understood with regards to etiology (Rayner et al., 2016). Previously, farmers identified creeping smothers in over 40% of their flocks (Barrett et al., 2014), and reported that once the behavior begins, it recurs throughout the laying period (Bright and Johnson, 2011).

There have been few studies reporting piling behavior. Campbell et al. (2016) observed a large number of piling events in 2 flocks of ∼49,000 laying hens in tiered aviaries in the United States but recorded no smothering. In this case piling did not occur at a predictable time of day, however, in a UK case study we observed creeping piles to be predictable at a flock level, with time of day (midday) and location largely habitual (Herbert et al., 2021). As this finding was based on one flock, it is possible that this was an effect related to management. Indeed, the primary management strategy for creeping smothers is walking through the birds to disperse them (Barrett et al., 2014), and as such, the timing of piling behavior might be linked to the timing of these walk throughs. However, in a study of 27 UK flocks, of which 18 showed piling, Winter et al. (2022a) also found time of day effects, supporting midday as a peak time for piling behavior. Even with this relatively large sample size of piling flocks, there is likely to be an underrepresentation of the true prevalence of piling since flocks were only observed for 1 d and video footage did not cover the full area of the chicken house. Piling events were also frequently seen in 13 Swiss flocks, observed for 1 d at 20 and 30 wk of age (Winter et al., 2021). Although definitions of piling differ between studies and there is a suggestion that piling behavior presents differently across white and brown hybrids, taken together these results suggest piling is a frequent behavior performed by laying hens.

Piling can vary in duration and severity, including the density of the pile and the number of birds in the pile. In Herbert et al. (2021) we observed piling events with 188 birds/m^2^ and up to 1,204 birds in 1 pile. Piling in such great numbers and density for any duration is likely to result in an increase in the body temperature of birds and increase the risk of injury. Thus, when we reviewed why hens pile in Gray et al. (2020) we suggested that piling could have welfare impacts not related to smothering deaths and proposed the following hypothesis: “recurrent piling events will negatively impact animal welfare through one or more of the following: i) heat stress; ii) physical injury; iii) stress.” It is feasible that piling causes delayed deaths, rather than at the time of the smother. We also proposed that smothering could impact production through egg quality as a secondary impact of stress, heat stress, physical contacts, or physical injury (Gray et al., 2020). There was no evidence of an effect of piling severity on production within the case study observed in Herbert et al. (2021), however, this case study was of only one flock making comparisons of production difficult.

In this study we observed piling behavior of 13 flocks of UK free-range hens across a 3-wk period. The main aims were to assess when piling was most common and to pair this with commercially gathered data to evaluate the association between piling and the production and mortality of commercial free-range flocks. To explore this aim we tested four hypotheses:

H1: Higher numbers of piling instances and longer total piling duration in a flock will be associated with a reduction in overall production of a flock (as measured by number of eggs) the following day.
H2: Higher numbers of piling instances and longer total piling duration in a flock will be associated with an increase in the number of Grade B eggs (unsuitable for sale as shell eggs) the following day.
H3: Higher numbers of piling instances and longer total piling durations in a flock will be associated with higher flock mortality.
H4: The most common time for piling to occur will be the period around midday.

We preregistered our hypotheses and methods on the Open Science Framework (https://osf.io/5vq2g/). To generate new hypotheses for future research, we also show exploratory results describing aspects related to differences in piling severity within- and between-flocks.

## MATERIALS AND METHODS

This study was approved by the Newcastle University Animal Welfare Ethical Review Board (Ref: 839). Video was recorded in 13 commercial free-range laying hen flocks, of which 3 were organic. Camera systems were placed opportunistically for flocks known to be piling. We attempted to match the characteristics (age, breed, system type) of piling flocks to film similar flocks reported not to be piling. The flocks were located on 7 farms, all in the county of Cumbria in Northwest England and all participating producers gave consent for data collection. All flocks were housed in a flat deck production system, but other characteristics of the flocks varied. Flock sizes ranged between 3,000 and 16,000 birds and ages between 16 and 58 wk at camera placement. The birds were of two different hybrids, Lohmann Classic and Shaver Brown. Details for each flock can be found in Table 1. As recordings were taken at different times of year, footage from the period between October 2021 and September 2022 was selected for use in the study.

**Table 1:** An overview of the filmed flocks and their piling behavior.

Flock ID	No. of cameras	Total litter area of barn (m^2^)	Hybrid	Age at start of analysis (wk)	Analysis window	Flock size at start of analysis	Number of mortalities over analysis period	Total pile duration (min)	Total no. piles	Mean piles per day	Mean pile duration (min)	Total smother events	Mean pile size (number of birds)
1	5	289.8	SB	59	26 Oct–15 Nov 21	4750	30	3337	57	2.71 ± 1.39	57.00 ± 30.00	0	392 ± 187
2	4	497.56	SB	57	14 Oct–03 Nov 21	11,144	532^1^	469	14	0.67 ± 0.99	36.08 ± 32.60	8	404 ± 245
3	4	450	SB	57	14 Oct–03 Nov 21	10,970	461	241	4	0.19 ± 0.39	60.25 ± 16.75	9	263 ± 22
4	4	450	LC	19	25 Jul–14 Aug 22	11,998	67	4920	115	5.48 ± 1.59	42.05 ± 35.56	8	349 ± 186
5	6	268.61	SB	21	06 Jul–26 Jul 22	2924	5	2963	153	7.29 ± 4.03	19.49 ± 12.97	1	358 ± 180
6	6	267.01	SB	21	06 Jul–26 Jul 22	2996	4	2382	105	5.00 ± 2.09	23.13 ± 20.49	0	335 ± 189
7	6	910	SB	18	06 Jul–26 Jul 22	15,996	62	6441	173	8.24 ± 2.97	36.60 ± 25.57	2	465 ± 329
8	6	910	SB	18	06 Jul–26 Jul 22	15,999	208	7320	185	8.81 ± 3.62	38.94 ± 26.67	4	431 ± 299
9	4	728	SB	72	05 Jan–25 Jan 22	14,748	73	3985	29	1.38 ± 0.90	137.41 ± 107.63	2	493 ± 218
10	4	728	SB	28	16 May–05 Jun 22	15,927	11	3697	52	2.48 ± 1.47	68.46 ± 53.08	2	511 ± 233
11	6	767.6	SB	59	26 Aug–15 Sep 22	14,824	146	6081	89	4.24 ± 1.57	67.57 ± 57.61	0	291 ± 238
12	6	337.5	LC	30	15 Mar–04 Apr 22	7590	7	9953	172	8.19 ± 2.59	57.87 ± 32.44	2	296 ± 238

1 Two hundred and forty birds removed from flock on 28/10/2021 included in mortality figure. Abbreviations: LC, Lohmann Classic; SB, Shaver Brown.

The video recordings were taken using Hikvision DS-2CD2346G2-IU cameras connected to Hikvision DS-7608NI-K2/8P recorders. Most sheds were furnished with 6 cameras, 3 spaced evenly on either side of the shed over the litter pointing down to the floor for an overhead view (systems with 4 cameras had 2 evenly spaced on either side of the shed, and the 1 with 5 a 2 and 3 split). The litter area covered by each camera varied according to the height of the shed's roof, which governed the height the cameras were installed at, and the angle that the cameras were set at. Accordingly, each camera covered a different area of litter, however all cameras covered enough litter to establish whether piling was or was not present (example screenshots are provided in the supplementary materials).

The recorder was set to capture the period between 0800 and 2000, which approximately corresponded to the lighting schedules of the various farms and allowed for more efficient use of limited storage space. One flock's footage was found to be unsuitable due to the angle of the cameras and this flock was discounted from the dataset.

Video footage was assessed for piling behavior for all camera locations within the included 12 flocks, totaling 64 cameras. Twenty-one days’ worth of videos were assessed for each of the 64 cameras (some videos were missing at random, details in Table 2). For all flocks the dates of the video analyzed were chosen so that they corresponded as closely as possible with the 10 d prior to the first smother recorded in the production data, the day of the first recorded smother, and the 10 d following the first recorded smother. For flocks where smothering was not recorded the dates selected were based on the start of a period of 3 wk of quality footage where cameras had been previously installed (see https://osf.io/5vq2g/ for a breakdown table).

**Table 2:** A summary of missing video footage.

Flock	D	Period missing	Number of cameras affected	Number of hours missing
Flock 3	1	1900–2000	1	1
Flock 3	2	1800–2000	1	2
Flock 3	2	1900–2000	2	2
Flock 3	3	1900–2000	2	2
Flock 3	4	1900–2000	3	3
Flock 3	12	0800–2000	1	12
Flock 5	3	0800–1400	1	6
Flock 5	17	1900–2000	1	1
Flock 5	18	1400–2000	1	6
Flock 5	18	1700–2000	1	3
Flock 5	18	1900–2000	1	1
Flock 6	18	1900–2000	1	1
Flock 6	19	0800–2000	1	12
Flock 6	20	0800–2000	1	12
Flock 6	21	0800–2000	1	12
Flock 9	2	1300–2000	4	28
Flock 9	3	0800–1400	4	24
Flock 9	4	1000–2000	1	10
Flock 9	5	0800–2000	1	12
Flock 9	7	0900–2000	1	11
Flock 9	8	0800–2000	1	12
Flock 9	9	0800–1000	1	2
Flock 10	2	1900–2000	1	1
Flock 10	13	1400–2000	1	6
Flock 10	17	0900–1000	1	1
Flock 10	19	0800–0900	1	1

Piling was defined as follows, adapted from Herbert et al. (2021): at least 30 birds aggregated in the closest possible proximity (overlapping body outlines) for at least 1 min while performing no other discernible behaviors (e.g., dustbathing), but they may be moving. Piling was recorded when all the conditions of our definition were met and ceased when any of them failed. Observations were conducted by skipping through 10 min of footage at a time. This method was chosen to process a larger volume of footage, but as a result smaller, shorter piles may have been missed. To combat this, any increase in numbers or activity of the birds between skips was investigated for a piling event in the intervening 10 min. Upon finding a pile in the video footage the following was carried out:

1. The time of the pile formation was discerned
2. Any attributable cause was noted
3. The video was then scanned to find the time of dispersal and any cause noted
4. The period of the pile was scanned to find its peak and to note an estimate of the number of birds involved

This method was formulated by DA who then trained BS and HN. The number of piling events and total duration of piling events for each flock on each day was pooled from the video observations. The estimate of the number of birds in a pile was informed by a manually counted screenshot of a pile which had a grid overlaid onto it. Each pile that was fully within the camera's view at its peak was then judged according to the number of grid-squares it filled. With experience researchers were able to estimate without the use of the grid. Production figures (egg numbers, Grade B eggs, and mortality) were accessed using a web portal system called BirdBox which is used by the management of all the farms to track figures (https://www.faifarms.com/birdbox/). BirdBox collates data on a wide range of variables that are useful in managing the farms day to day. These include environmental data such as temperature, carbon dioxide levels, and light intensity inside the shed, production data including number of eggs, farm seconds, and eggs per bird, husbandry data with the likes of bird weight, food consumption and water consumption, and management data such as feed bin fullness, litter checks, and veterinary treatments.

Production figures were matched to the dates of video observations for each flock.

### Statistical Analysis

All analyses and data visualization were conducted using R v. 4.2.1. Models were computed using the Stan programming language via the brms package (version 2.12.), which estimates parameters using Hamiltonian Monte Carlo. Four Markov chains were run, each with a warm-up period of 2,500 iterations and 5,000 iterations used for sampling. Thinning was set to 1. Convergence was checked using the Gelman-Rubin statistic with convergence indicated by values close to 1 and less than 1.05.

Hypothesis 4, the timing of the piling events was not formally tested but descriptive results were calculated. These descriptive results were achieved by manipulating the data into a binary form of pile or no pile. Every pile regardless of length was given a score of 1, then the number of piles per timepoint (timepoints were split into hours, i.e., 0800–0859, 0900–0959, etc.) were collated and these data were used to form histograms which showed the pile distributions across the 0800 to 2000 period studied.

Linear mixed-effect models were used to test hypotheses 1, 2, and 3. The priors for the effects of day on egg production and mortality were informed by breed standards and were set using student *t* distributions with parameters as follows: egg production (df = 3, mean = −0.4, SD = 5); mortality (df = 3, mean = −0.2, SD = 1). When analyzing egg metrics, we excluded flocks under 21 wk of age (*n* = 3) such that we could ensure that the analyzed flocks had come into lay. Specific details of datasets used for each analysis can be found under the relevant model equations. Data were missing for 184 h (1.14% of the dataset). Data were analyzed at the day level, and as there were no full days of missing data, data did not need to be imputed. Predictor variables were checked for multicollinearity using the variance inflation factor.

Analysis code is available at https://osf.io/5vq2g/ but reporting the linear model equations below means that the analysis is replicable even if R syntax changes over time.

H1: Higher numbers of piling instances and longer total piling duration in a flock will be associated with a reduction in overall production of a flock (as measured by number of eggs) the following day.
  $$\text{Egg}s_{F\left[i\right]}∼\text{Poisson}\left(\lambda_{F\left[i\right]}\right)$$
  $$\text{log}\left(\lambda_{F\left[i\right]}\right)\;=\;\alpha_{1}\;+\;\beta_{1}N_{F\left[i-1\right]}\;+\;\beta_{2}T_{F\left[i\right]}+\;\text{log}\left(S_{F\left[i\right]}\right)\;+\;r_{F}$$

The number of eggs produced in flock *F*, on day *i*, is a function of fixed effects of the number of piling events on the previous day ($\beta_{1}N_{F[i-1]})$ and the time (day of video observation: $\beta_{2}T_{F[i]}$), which accounts for any changes in production over the 3-wk period. There is an offset variable for the size of the flock ($\text{log}(S_{F[i]})$) and a random effect of flock ($r_{F})$. This analysis was carried out using data from flocks of over 21 wk of age (to ensure birds had started laying), on both piling and non-piling days.

$$\text{Egg}s_{F\left[i\right]}∼\text{Poisson}\left(\lambda_{F\left[i\right]}\right)$$

$$\text{log}\left(\lambda_{F\left[i\right]}\right)\;=\;\alpha_{1}\;+\;\beta_{1}D_{F\left[i-1\right]}\;+\;\beta_{2}T_{F\left[i\right]}+\;\text{log}\left(S_{F\left[i\right]}\right)\;+\;r_{F}$$

To assess the effect of duration of piling events, we used data from flocks of over 21 wk (to ensure birds had started laying), only on piling days. The model is as above, with the only change being the duration of piling events on day *i* − 1 ($\beta_{1}D_{F[i-1]})\;$replaces the number of piling events.

H2: Higher numbers of piling instances and longer total piling duration in a flock will be associated with an increase in the number of Grade B eggs (unsuitable for sale as shell eggs; also known as seconds) the following day.
  $$\text{Second}s_{F\left[i\right]}∼\text{Poisson}\left(\lambda_{F\left[i\right]}\right)$$
  $$\text{log}\left(\lambda_{F\left[i\right]}\right)\;=\;\alpha_{1}\;+\;\beta_{1}N_{F\left[i-1\right]}\;+\;\beta_{2}T_{F\left[i\right]}+\;\text{log}\left(E_{F\left[i\right]}\right)\;+\;r_{F}$$

The number of seconds produced in flock *F*, on day *i*, is a function of fixed effects of the number of piling events on the previous day ($\beta_{1}N_{F[i-1]})$ and the time (day of video observation: $\beta_{2}T_{F[i]}$), which accounts for any changes in production over the 3-wk period. There is an offset variable for number of eggs produced ($\text{log}(E_{F[i]})$) and a random effect of flock ($r_{F})$. This analysis was carried out using data from flocks of over 21 wk (to ensure birds had started laying), on both piling and non-piling days.

$$\text{Second}s_{F\left[i\right]}∼\text{Poisson}\left(\lambda_{F\left[i\right]}\right)$$

$$\text{log}\left(\lambda_{F\left[i\right]}\right)\;=\;\alpha_{1}\;+\;\beta_{1}D_{F\left[i-1\right]}\;+\;\beta_{2}T_{F\left[i\right]}+\;\text{log}\left(E_{F\left[i\right]}\right)\;+\;r_{F}$$

To assess the effect of duration of piling events on seconds produced, we used data from flocks of over 21 wk (to ensure birds had started laying), only on piling days. The model is as above, with the only change being the duration of piling events on day *i* − 1 ($\beta_{1}D_{F[i-1]})\;$replaces the number of piling events.

H3: Higher numbers of piling instances and longer total piling durations in a flock will be associated with higher flock mortality.
  $$\text{Mortalit}y_{F\left[i\right]}∼\text{Poisson}\left(\lambda_{F\left[i\right]}\right)$$
  $$\text{log}\left(\lambda_{F\left[i\right]}\right)\;=\;\alpha_{1}\;+\;\beta_{1}N_{F\left[i-1\right]}\;+\;\beta_{2}T_{F\left[i\right]}+\;\text{log}\left(S_{F\left[i\right]}\right)\;+\;r_{F}$$

The number of birds in flock *F*, which died on day *i*, is a function of fixed effects of the number of piling events on the previous day ($\beta_{1}N_{F[i-1]})$ and the time (day of video observation: $\beta_{2}T_{F[i]}$), which accounts for any changes in production over the 3-wk period. There is an offset variable for the size of the flock ($\text{log}(S_{F[i]})$) and a random effect of flock ($r_{F})$. This analysis was carried out using data from all flocks for days where there are no smothering events, including piling and nonpiling days. This allows for deaths occurring as a nondirect result of piling to be analyzed.

$$\text{Mortalit}y_{F\left[i\right]}∼\text{Poisson}\left(\lambda_{F\left[i\right]}\right)$$

$$\text{log}\left(\lambda_{F\left[i\right]}\right)\;=\;\alpha_{1}\;+\;\beta_{1}D_{F\left[i-1\right]}\;+\;\beta_{2}T_{F\left[i\right]}+\;\text{log}\left(S_{F\left[i\right]}\right)\;+\;r_{F}$$

The model is as above, with the only change being the duration of piling events on day *i* − 1 ($\beta_{1}D_{F[i-1]})\;$replaces the number of piling events. This analysis was carried out using data from all flocks for days where there are no smothering events, but where there are piling events.

## RESULTS

The final dataset comprised 252 d of data across 12 flocks, amounting to approximately 15,372 h of video of which 184 h were missing at random due to equipment failure (Table 2). We recorded 1,148 individual piles across 216 d, and piling was noted in 100% of flocks. The mean pile duration was a little over 45 min (45.28 ± 42.13 min; minimum = 1 min; maximum = 422 min), and on average the flocks piled more than 4 times per day (4.60 ± 3.71 piles per d; minimum = 1; maximum = 18). The mean number of birds involved in recorded piles that were able to be estimated was approximately 381 (380.65 ± 253.30; minimum = 30 birds; maximum = 1,500 birds). During the study period 75% of the flocks (9/12 flocks) had a smother event but not all of these were recorded on video (these smothering data were recorded by stock people and accessed via BirdBox). There was a total of 38 smothers: 25 of these were on days where piling was caught on camera, and 13 when no piling was seen on camera. For all our results, we therefore acknowledge that there may be a discrepancy between the number of piles observed and the true number of piling events. This is due to cameras not covering all the litter area and due to some sheds having more cameras than others (see Table 1). As there was the possibility that flocks with fewer cameras per litter area may have fewer piles observed, we checked for this visually. Plotting the area of litter per camera against the total number of observed piles, we saw no linear relationship (Figures S1 and S2), and as such were confident in using the number of piles as a predictor.

The results presented below are model estimates and summary statistics, reported to 2 significant figures. Model parameters are summarized by the mean and 95% highest density interval (**HDI**; the 95% most likely values in the distribution). Significance is inferred when the highest density interval does not contain zero. No model showed multicollinearity on the predictor variables (largest value = 1.026). All raw data are available as supplementary material and at https://osf.io/5vq2g/.

H1: Higher numbers of piling instances and longer total piling duration in a flock will be associated with a reduction in overall production of a flock (as measured by number of eggs) the following day.

### Eggs by Number of Piles

**Figure 1:**
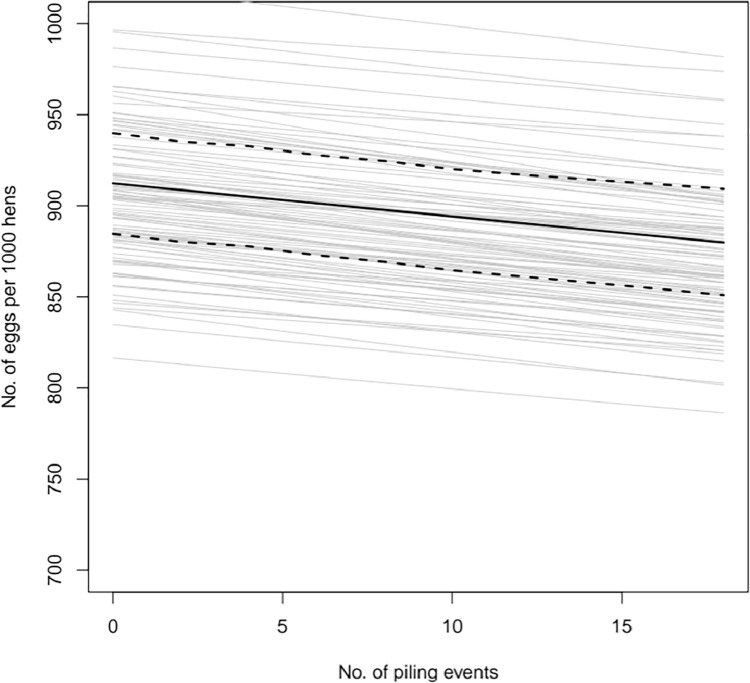
The effect of the number of piling events on the number of eggs produced on the following day. The posterior mean is denoted by the solid black line with the 95% highest density interval of the mean shown by the dashed lines. The gray lines show 100 representative samples from the posterior distribution marginalizing across flock random effects.

Overall, there was an average of 912 eggs (95% HDI 881–940) per 1,000 hens. We found a significant association between both the number of piling events and day with the number of eggs (Figure 1). As the number of piles increased by 1, the eggs produced decreased by a multiplicative factor of 0.998 (95% HDI 0.997–0.999). For our average of ∼4 piles per day, this translates as a loss of 7.35 (3.76–10.93) eggs per 1,000 birds. As the day of observation increased by 1, the number of eggs increased by a multiplicative factor of 1.00042 (HDI 1.00017–1.0068), equating to an extra 1.53 (0.62–2.48) eggs per week, per 1,000 birds.

### Eggs by Duration of Piles

The model estimated an average of 906 eggs (95% HDI: 882–930) per 1,000 hens. There was no significant effect of the total duration of daily piling events on the number of eggs produced (Table S1). We found a significant association of day: as day increased by 1, the number of eggs increased by a multiplicative factor by 1.00033 (95% HDI: 1.00005–1.00061), equating to an extra 2.09 (0.32–3.87) eggs per week, per 1,000 birds.

H2: Higher numbers of piling instances and longer total piling duration in a flock will be associated with an increase in the number of Grade B eggs (unsuitable for sale as shell eggs) the following day.

### Grade B by Number of Piles

The number of seconds was estimated at 25 (95% HDI 15–39) per every 1,000 eggs. There was no significant effect of either the total duration of daily piling events or the day on the number of Grade B eggs produced (Table S2).

### Grade B Eggs by Duration of Piles

**Figure 2:**
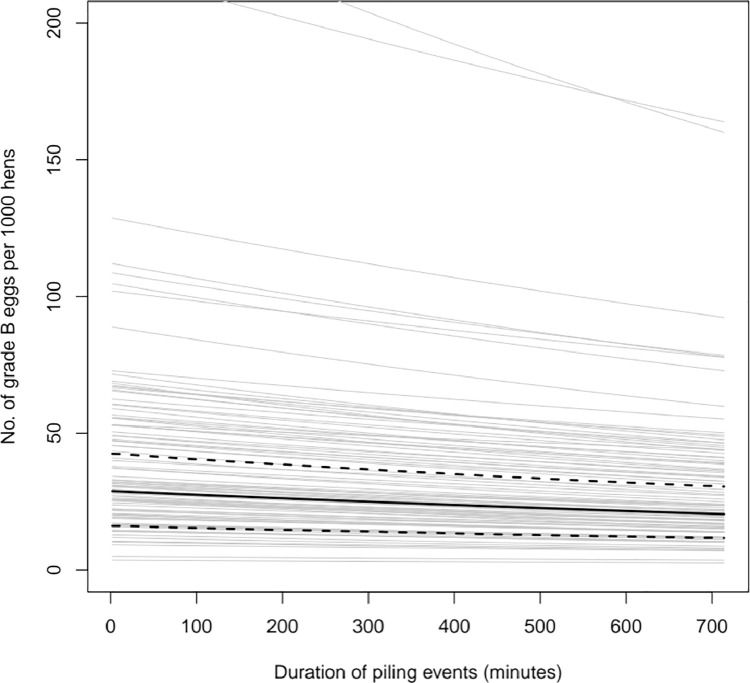
The effect of the total piling duration on the number of Grade B eggs produced on the following day. The posterior mean is denoted by the solid black line with the 95% highest density interval of the mean shown by the dashed lines. The gray lines show 100 representative samples from the posterior distribution marginalizing across flock random effects.

There was an average of 28 Grade B eggs (95% HDI 18–44) for every 1,000 eggs. As piling duration increased by 15 min, Grade B eggs per 1,000 eggs decreased by a multiplicative factor of 0.993 (0.991–0.994), translating as a decrease of 0.82 (0.63–1.00) Grade B eggs for every 1 h increase in piling duration (Figure 2). There was no significant effect of day (Table S3).

H3: Higher numbers of piling instances and longer total piling durations in a flock will be associated with higher flock mortality.

### Mortality by Number of Piles

Nonsmothering-related mortality was low, estimated at 0.10 counts of mortality per day, per 1,000 birds (95% HDI: 0.04–0.23). There was no significant effect of either the number of piling events or the time (Table S4).

### Mortality by Duration of Piles

For days when there were piling events, the model estimated 0.074 mortality counts per day, per 1,000 birds (95% HDI: 0.031–0.17). There was no significant effect of the total duration of piling events (Table S5). We found that as day increased by 1, mortality increased by a multiplicative factor of 1.025 (1.0059–1.044). Due to the very low estimated mortality, this equates only to an increase of 0.0018 (0.00042–0.0031) counts per 1,000 birds, for a 1 unit increase in day.

H4: The most common time for piling to occur will be the period around midday.

**Figure 3:**
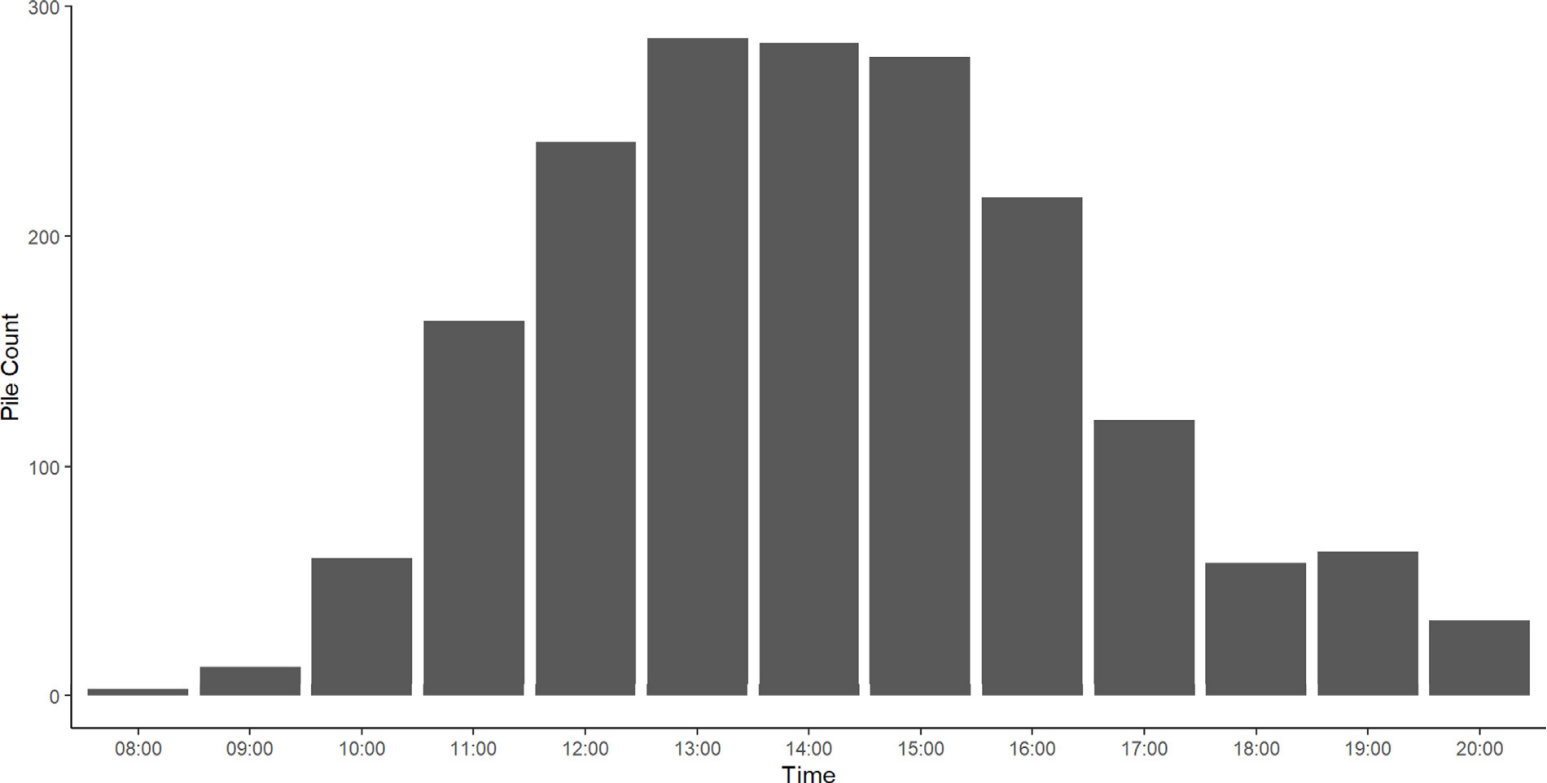
The total number of piling events recorded throughout the day, across flocks. Note that the same piling event can appear in multiple time bins.

More piles were found to have occurred in the time between 1300 and 1359 than at any other time (Figure 3) with around 25% (286/1,158) of all recorded piles falling within this period, closely followed by 1400 to 1459 (∼25%, 284/1,158) and 1500 to 1559 (∼24%, 278/1,158). It is important to note that piles may span time periods and as such the same pile may be counted in multiple time bins.

**Figure 4:**
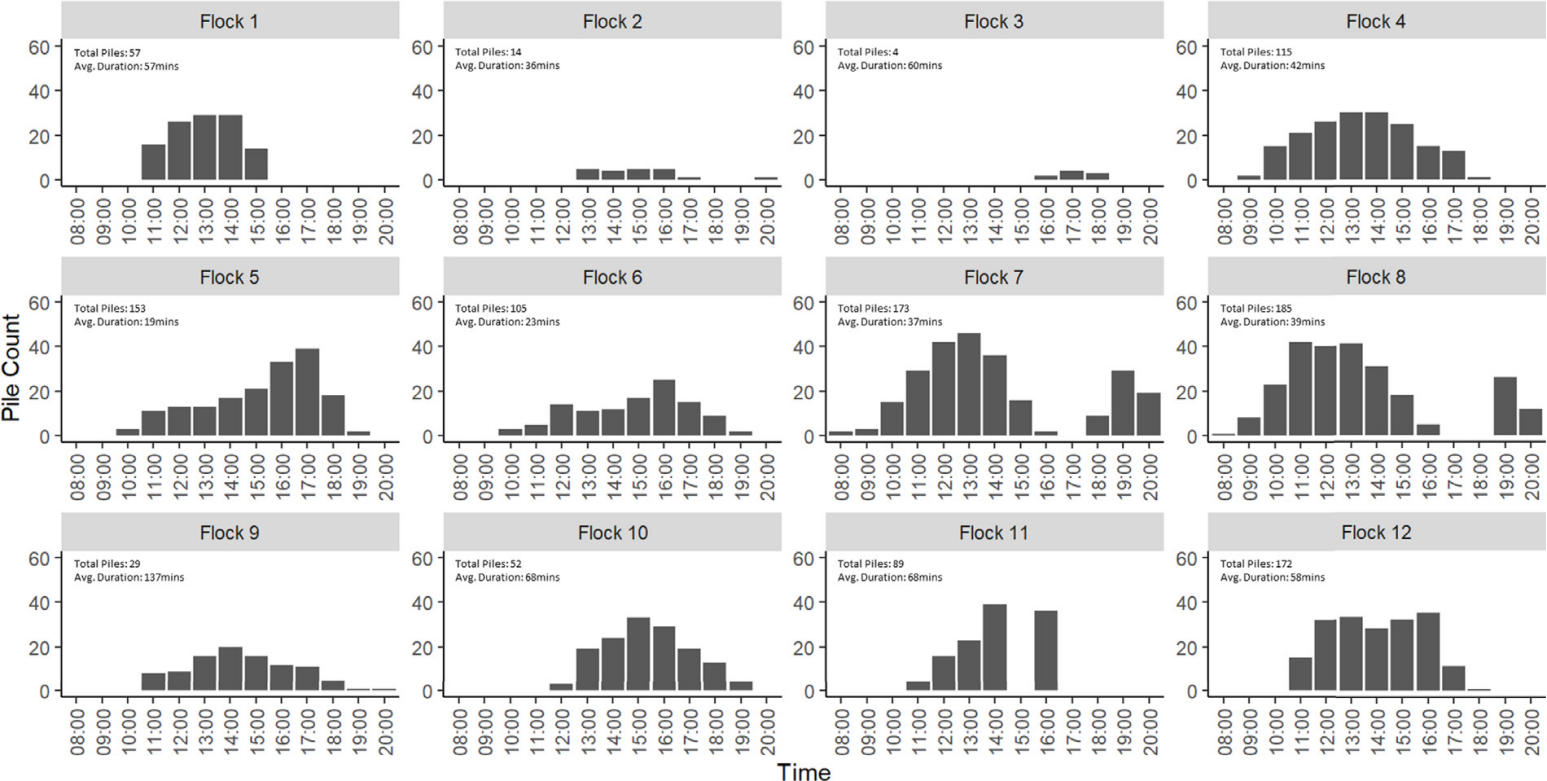
The total number of piling events recorded throughout the day, for each flock. Note that the same piling event can appear in multiple time bins. Avg. refers to the mean duration.

At individual flock level the time of peak numbers and duration of piles was highly variable (Figure 4). The modal peak time for numbers of piles at individual flock level is 1300 to 1359 which contained the highest number of individual piles in 33% of flocks (4/12). There are 3 modal peak times for duration of piles however, 1300 to 1359, 1400 to 1459, and 1700 to 1759, with each of these being the peak time in 25% of flocks (3/12). It is interesting to note that the distributions are also similar for sister flocks (those that are from the same hatch/rearing site) such as Flock 7 and Flock 8, and flocks that were housed in the same sheds but at different times such as Flock 9 and Flock 10.

### Exploratory Results

In addition to formally testing our predefined hypotheses, we also used our dataset to explore some other potentially interesting relationships to inform future work and generate new testable hypotheses. These are summarized in Table 3, with further information available in Supplementary material (Figures S3–S6). We stress that these relationships may be spurious, as we do not necessarily have the power to decipher true associations from flock-level factors.

**Table 3:** Additional findings.

Variables	Relationship
Age	Younger birds potentially pile more often but older birds pile for longer whenever they do.
Season	Higher number of piling events in summer months.
Flock size	Flocks of over 12,000 birds had bigger piles than both flocks of fewer than 6,000 and flocks of between 6,000 and 12,000 birds.
Breed	Shaver Brown birds may pile in larger numbers than Lohmann.

## DISCUSSION

This paper provides the first quantification of the impact of piling events on laying hen production, adding much needed data to an understudied behavior. We originally set out to compare piling vs. nonpiling flocks but interestingly all recorded flocks showed piling behavior with peak piling time occurring around 1 to 2 pm. This was an interesting result and provides support for the idea that piling behavior is much more prevalent in UK flocks of brown hens than is evident from records of smothering deaths alone. We found that higher numbers of piling events were associated with poorer egg numbers the next day, with approximately a 0.7% reduction in production. Using the current egg price from the British Free Range Egg Producers Association (£1.40 free range and £2.19 organic; accessed on 03/04/23), and assuming 300 piling days over the flock cycle, this equates to economic losses of £1,202.31 and £4,028.05 for flocks of 3,000 organic birds and 16,000 free-range birds, respectively. Although we found a significant association between piling duration and Grade B egg numbers, the effect size was very small with a decrease of less than 1 Grade B egg for every 1 h increase in piling duration. Below, we discuss potential explanations relating to each of our hypotheses and highlight where further research is needed.

### Higher Numbers of Piling Instances and Longer Total Piling Duration in a Flock Will Be Associated With a Reduction in Overall Production of a Flock (As Measured by Number of Eggs) the Following Day

We found that the number of piling events, but not the total duration of piling events, was associated with a decrease in the number of eggs produced on the following day. However, due to the simplicity of our models and the relatively small sample size, we are careful not to infer causality of the association. The association may be linked to either a direct impact of the piling on production or an indirect link between stress and both piling and production, and we address each of these below.

First, given that the density of birds in a pile has been estimated to reach 184 birds/m^2^ (Herbert et al., 2021) we hypothesized in a previous paper that piling may lead to heat stress (Gray et al., 2020). Heat stress can affect egg quality, egg production (Mashaly et al., 2004), ovarian function (Rozenboim et al., 2007), and feed intake (Barrett et al., 2019). More research is needed into the temperature reached inside a pile, however due to the close proximity of the birds we believe that both the cumulative effect of their body heat and the inability to perform behaviors linked to cooling (i.e., wing spreading) could contribute to high temperatures capable of leading to heat stress. We believed that an increased duration of piling would alter production due to prolonged exposure to higher temperatures. However, our data do not support this. It may be that the number of piling events is more salient than duration as there is the potential for a higher proportion of the flock to be involved. A further explanation is that the speed of temperature transition may be more impactful than the overall exposure to high temperature. For example, Kang et al. (2020) found that mortality was higher for chickens undergoing rapid changes in temperature than those experiencing gradual transition. To further explore the relationship, future work should concentrate on understanding the temperature and humidity levels reached in piling events, and the speed at which chickens involved in piling events experience ambient temperature changes.

Second, there may be an indirect link between piling and production via a common cause, such as stress. It may be that, in parallel, stressed birds are more likely to be involved in piling incidents and that stress also contributes to poorer production. The link between stress and poor production is fairly well documented. For example, Sokół et al. (2019) found a strong relationship between CORT levels (used as a proxy of stress) and egg production in a commercially relevant breed of laying hen, whereby birds with higher CORT levels produced fewer eggs. Increased CORT is also evident in birds which have been reared in higher density environments (von Eugen et al., 2019), which is of relevance to piling behavior.

However, to address whether stressed birds are more likely to be involved in piling incidents, there is a need to disentangle the direction of the association. Is piling itself causing stress or are stressed birds more likely to pile? We have discussed the potential stress implications of piling in a previous review (Gray et al., 2020) but the idea that stressed birds may have a higher propensity to pile needs further consideration. It may be that piling is an exaggerated form of huddling, or that it is a response to a perceived threat. Huddling for thermal comfort and for social buffering tends to be a static behavior, with groups of animals clustering to maintain heat or gain social contact, whereas the piling we observe in laying hens often results in a slow, vortex-like collective movement. Hens are often seen pushing in between other birds, moving closer toward the center of the pile. This suggests some form of attraction or repulsion. At the formation of an initial cluster, this may be due to social attraction with hens moving toward a group of conspecifics. However, once the pile reaches a certain size, given the unlikeliness that birds can see the middle, it may be more probable at this stage that hens are moving away from the edge of the crowd. This is seen in wild populations of birds, described by the marginal predation theory. If higher levels of stress in a flock increases an individual's perception of danger, then stressed flocks may be more likely to instigate piling events.

### Higher Numbers of Piling Instances and Longer Total Piling Duration in a Flock Will Be Associated With an Increase in the Number of Grade B Eggs

In conflict with our second hypothesis, we found longer piling durations to be associated with a reduction in Grade B eggs, rather than an increase in Grade B eggs. However, this was a very small effect, unlikely to have any economic impact.

There are numerous reasons for which an egg can be downgraded to Grade B, including if the eggs are dirty. Eggs are more likely to become dirty if they are laid outside of the nest boxes (known as “floor eggs”). Our initial thoughts were that a decrease in Grade B eggs could be due to a decrease in floor eggs. This may be because floor eggs are broken by piling hens, or due to an increase in the number of times the producer walks through the birds (to break up the piling), resulting in more frequent collection of floor eggs and therefore decreasing the likelihood of the eggs becoming dirty. However, the data do not seem to support this (Figure S7). Other reasons eggs can be downgraded include them having weak shells and cracks, blood on the inside of the egg, texture defects, and if they are too small or too large. Our result could therefore be due to there being a reduction in any combination of these defects, however we lack the detailed data to assess this.

### Higher Numbers of Piling Instances and Longer Total Piling Durations in a Flock Will Be Associated With Higher Flock Mortality

We hypothesized that higher numbers of piling instances and longer total piling durations in a flock would be associated with nonsmothering mortality but found no evidence for this. This may be because the conditions in the pile, namely heat, humidity and force, are not as severe as assumed.

It is interesting to note that given the number of piles observed (1,086), even the direct mortalities from smothering were relatively low (38 smothering events). In humans, the risk of negative consequences of crowding are impacted by 4 factors, abbreviated as FIST (Fruin, 1993): Force, Information, Space (both in relationship to individual density and larger scale architectural features) and Time (duration). The cause of death in human crowding is most commonly asphyxiation with the risk increasing when density exceeds 10 people per m^2^ (Lee and Hughes, 2005). Even accounting for hens’ smaller size and weight, hens can be observed piling at densities which far exceed limits considered safe for people (Herbert et al., 2021).

Nonsmothering mortality as a consequence of piling does not seem to be a concern. Our hypothesis was driven by the idea that the conditions experienced repeatedly in piling events may be detrimental to overall hen health, increasing risk of indirect death. To better understand the risks of piling, the combination of experienced force, density and duration are important areas for future research both in terms of the impact of piling on health and welfare and the risk of a pile becoming a smother.

### The Most Common Time for Piling to Occur Will Be the Period Around Midday

Our hypothesis of midday for peak pile time was chosen in line with previous literature (Herbert et al., 2021; Winter et al., 2022a). In another study Winter et al. (2022b) also observed midday to be the peak pile time in experimental settings showing the strength of this interaction. Our findings, however, showed a slightly later peak of 1300 to 1359. We did, however, observe most of the duration of piling to fall between the early to mid-afternoon period (1200–1559) which is consistent with previous work (Winter et al., 2021). Our findings also agree with the notion that piling is less common in the morning. Our earliest pile started at 0808 but this was not typical, and piling did not often begin before 1100. It is largely accepted that this is to do with the fact that hens will prioritize other behaviors in the morning period (Winter et al., 2021). Most hens will lay their eggs in the morning (Villanueva et al., 2017) meaning that a large proportion of the flock is on the system or in the nest boxes. Lohmann Brown hens spend an average of 88.9 min in the nest box zone per visit (Rufener et al., 2019), meaning a significant period spent away from the litter. Gregarious nesting, when hens choose to lay in nest boxes which are already occupied (Tahamtani et al., 2018), further detracts from the number of birds on the floor in the morning period. In addition to nesting, hens also have a greater propensity to feed in the morning (Geng et al., 2022). This is somewhat dictated by when feed is offered but all flocks in this study had their first feed between 0600 and 0830 which includes the 0600 to 0800 period previously recorded as having the longest duration of feeding (Geng et al., 2022). We also found that although piling tailed off toward 1800, it could still be present as late as 2000 which is later than has previously been reported.

Additionally, our results indicate that flock variation in piling time is high. From our limited data, we see that piling distributions of sister flocks (those that come from the same hatch and were reared together) are similar to one another and that distributions of flocks from the same shed/sharing husbandry staff also pile in a similar distribution. This finding shows the possible influence of genetics, early life experience, and husbandry as factors influencing piling and warrants future investigation.

## CONCLUSIONS

Here we have presented what we believe is the largest analysis of piling behavior to date in the scientific literature. However, even with a large number of hours of footage this research only considers the behavior of 12 flocks of laying hens, all of which are brown breeds reared in the United Kingdom. We tested 3 hypotheses which predicted that piles would decrease production and increase nonsmothering mortality and found some evidence that piling decreased overall production but was also associated with a small decrease in seconds. Compared to other highly prevalent welfare problems in laying hens, such as feather pecking and keel bone damage, piling is understudied (Gray et al., 2020) meaning there is a dearth of scientific literature to be able to contextualize these findings. However, if piling does reduce the number of eggs produced this provides additional rationale to further study this behavior. In our sample all flocks were piling even if there had been no previous history of smothering, suggesting the behavior may be more prevalent than commonly assumed. Demand for cage-free egg production systems is rapidly growing globally (marketdataforecast.com, accessed 24 May 2023) and piling is found in cage-free systems. Based on an estimated 8.1 billion laying hens globally (FAO Stat (no date), 2023) of which an estimated 84.2% are in cages (Guyonnet, 2022) and an annual 5% increase in cage-free market (Marketdataforecast.com, 2023), this would equate to an additional 340 million chickens in cage-free systems each year. As a result, piling and smothering are likely to become an increasing issue globally with stock people lacking experience of this problem behavior. Future research is needed to confirm hypotheses generated from this study related to the age-related differences in piling behavior, seasonal or temperature effects, flock size effects and potential for strain differences or heritability of piling behavior.

## Appendix Supplementary materials

Image, application 1

Image, application 2

## Footnotes

Supplementary material associated with this article can be found in the online version at doi:10.1016/j.psj.2023.102989.
