# Supplementary material for: The effect of piling behavior on the production and mortality of free-range laying hens
Source: Poult Sci. 2023 Aug 5;102(10):102989. doi: 10.1016/j.psj.2023.102989 (PMC10465951; doi:10.1016/j.psj.2023.102989)
Supplement: Supplementary file 1 [file mmc1.docx]

Supplementary materials


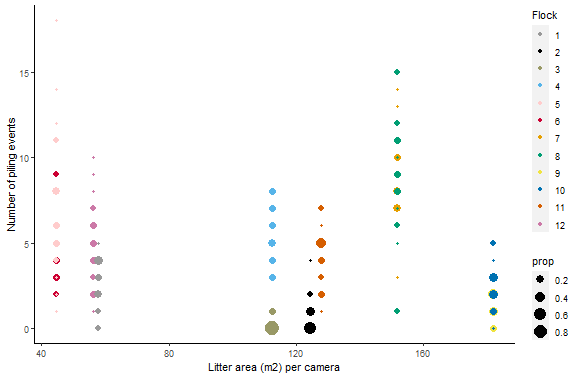


Figure S1. The effect of litter area per camera on the number of piling events observed per flock. Litter area per cameras was calculated as the total area of litter divided by the number of cameras in the shed. The size of the point is proportional to the number of observations.


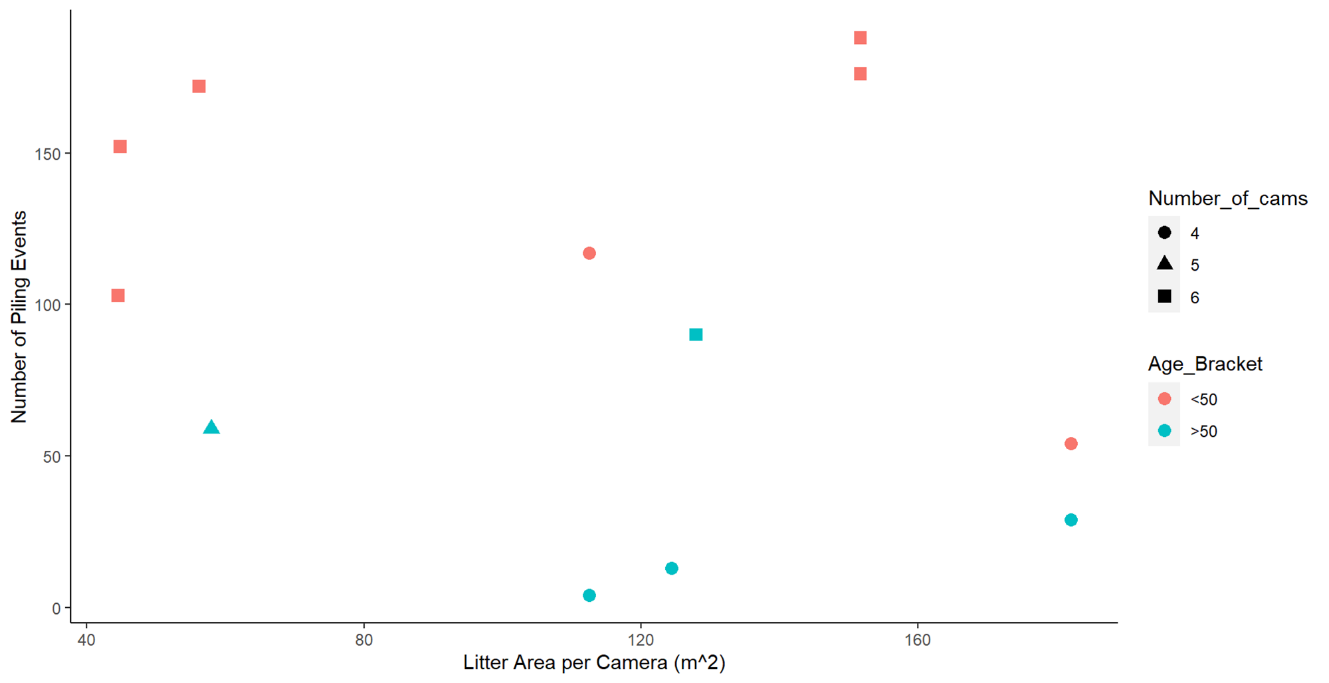


Figure S2. The effect of litter area per camera on the mean number of piling events observed per flock, split by age of flock. The number of piling events observed does not seem to be linearly related to the number of cameras in the shed.

Table S1 Summary of the effect of piling duration on the number of eggs produced the following day. All estimates are presented on the log scale.

|  | Estimate | 95% HDI |
| --- | --- | --- |
| Intercept | 6.80920 | 6.78186, 6.83544 |
| Duration of piling events | -0.00001 | -0.00023, 0.00021 |
| Day | 0.00033 | 0.00005, 0.00061 |

Table S2 Summary of the effect of the number of piling events on the number of grade B eggs produced the following day. All estimates are presented on the log scale.

|  | Estimate | 95% HDI |
| --- | --- | --- |
| Intercept | 3.21179 | 2.73720, 3. 66842 |
| Number of piling events | -0.00585 | -0.01204, 0.00037 |
| Day | 0.00170 | -0.000011, 0.00338 |

Table S3 Summary of the effect of the duration of piling events on the number of grade B eggs produced the following day. All estimates are presented on the log scale.

|  | Estimate | 95% HDI |
| --- | --- | --- |
| Intercept | 3.33477 | 2.85193, 3.79339 |
| Duration of piling events | -0.00732 | -0.00898, -0.00566 |
| Day | -0.00096 | -0.00284, 0.00095 |

Table S4 Summary of the effect of the number of piling events on the number of non-smothering mortalities. All estimates are presented on the log scale.

|  | Estimate | 95% HDI |
| --- | --- | --- |
| Intercept | -2.28318 | -3.22805, -1.45371 |
| Number of piling events | -0.00804 | -0.06871, 0.05293 |
| Day | 0.00405 | -0.01109, 0.01971 |

Table S5 Summary of the effect of the duration of piling events on the number of non-smothering mortalities. All estimates are presented on the log scale.

|  | Estimate | 95% HDI |
| --- | --- | --- |
| Intercept | -2.59885 | -3.46521, -1. 79557 |
| Duration of piling events | -0.00599 | -0.02215, 0.00977 |
| Day | 0.02387 | 0.00596, 0.04232 |

Exploratory results


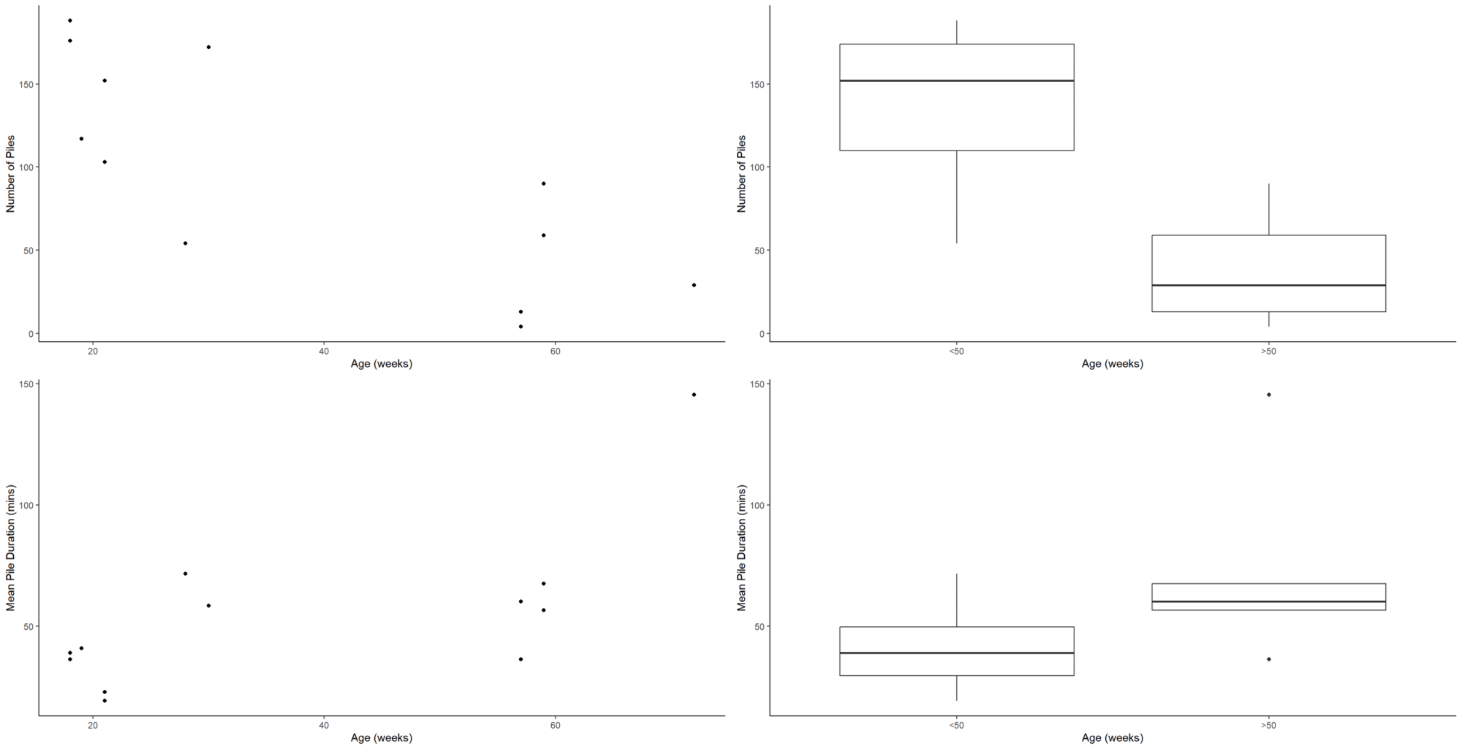


Figure S3. The effects of age on piling. Figure of 4 graphs the upper of which show the relationship between the number of piles and age, the lower of which show the relationship between the mean duration of piles and age. (<50 n = 7; >50 n = 5)


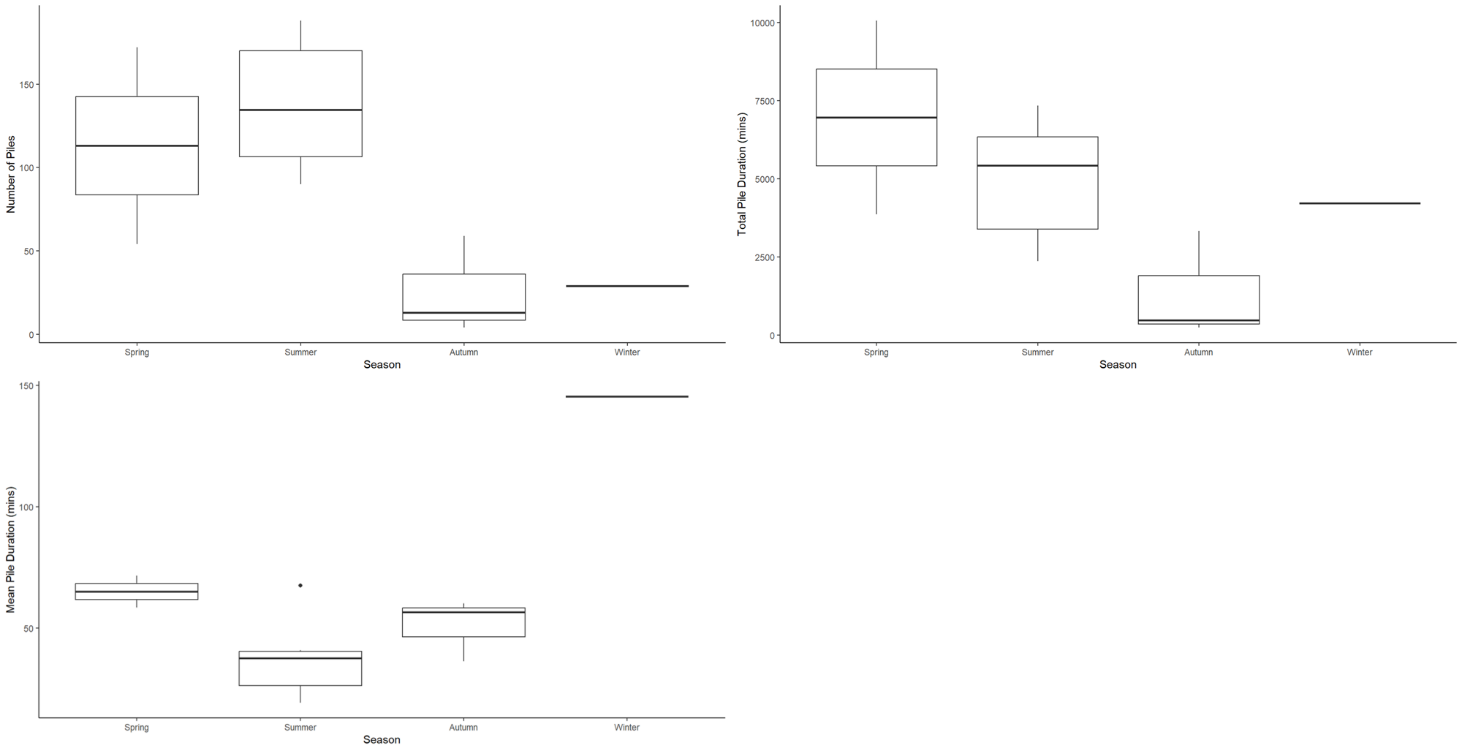


Figure S4, The effects of season on piling. Figure of 3 graphs the upper left of which shows the relationship between the number of piles and the season, the upper right shows the relationship between the total duration of piling per flock and the season, and the lower of which shows the relationship between the mean duration of piles and the season. (Spring n = 2; Summer n = 6; Autumn n = 3; Winter n = 1)


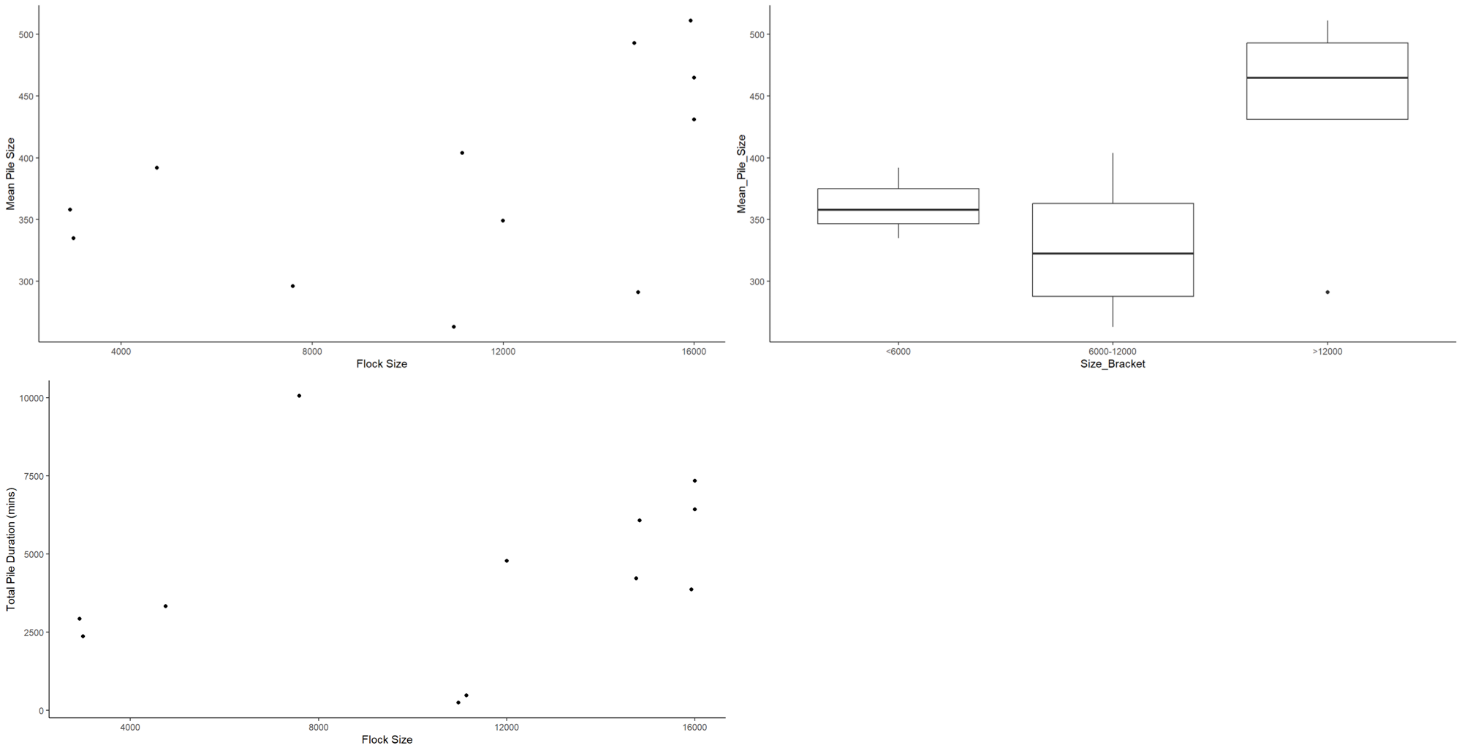


Figure S5, The effects of flock size on piling. Figure of 3graphs the upper of which show the relationship between the mean pile size and flock size, the lower of which shows the relationship between the total duration of piling and the flock size. (<6000 n = 3; 6000-12000 = 4; >12000 n = 5)


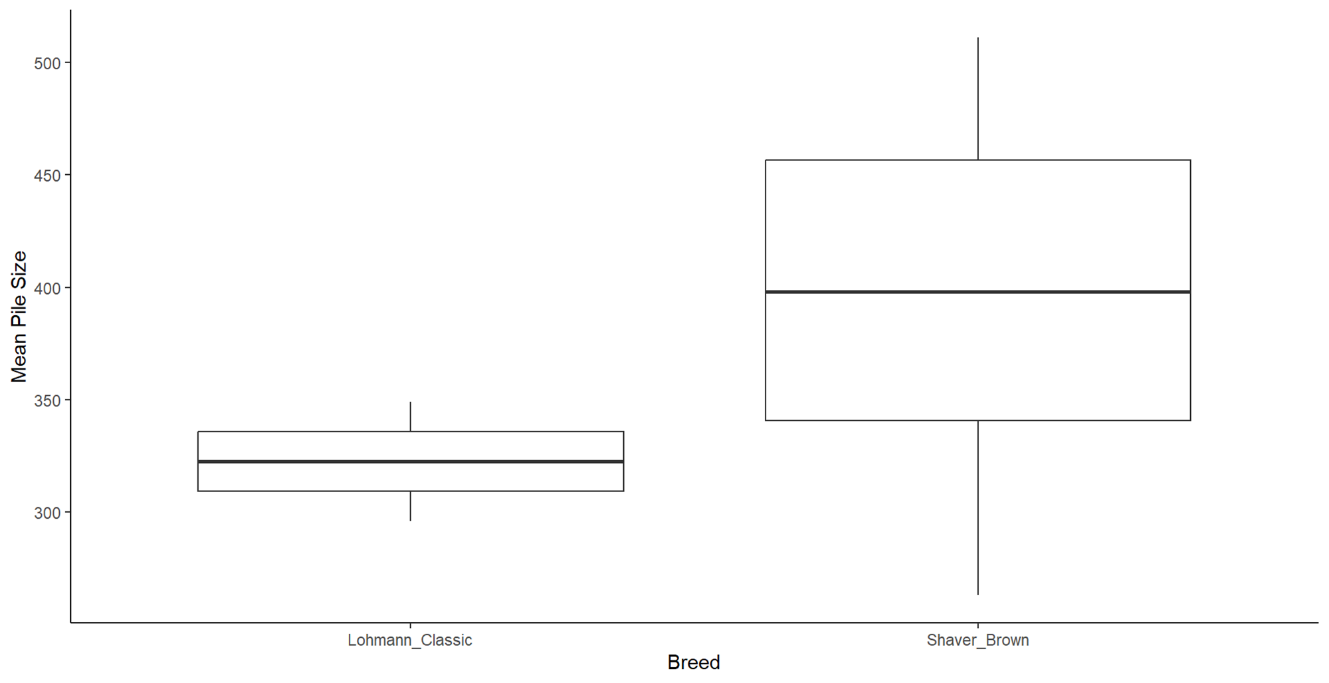


Figure S6, The effect of breed on piling. Figure shows the relationship between the mean pile size and the breed of chicken. (Lohmann Classic n = 2; Shaver Brown n = 10)


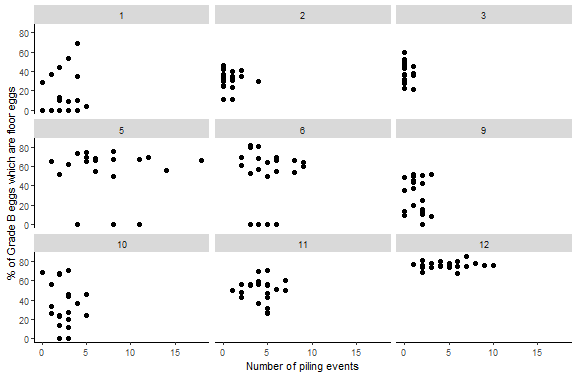


Figure S7 The association between the number of piling events per flock and the percentage of grade B eggs which are classified as floor eggs


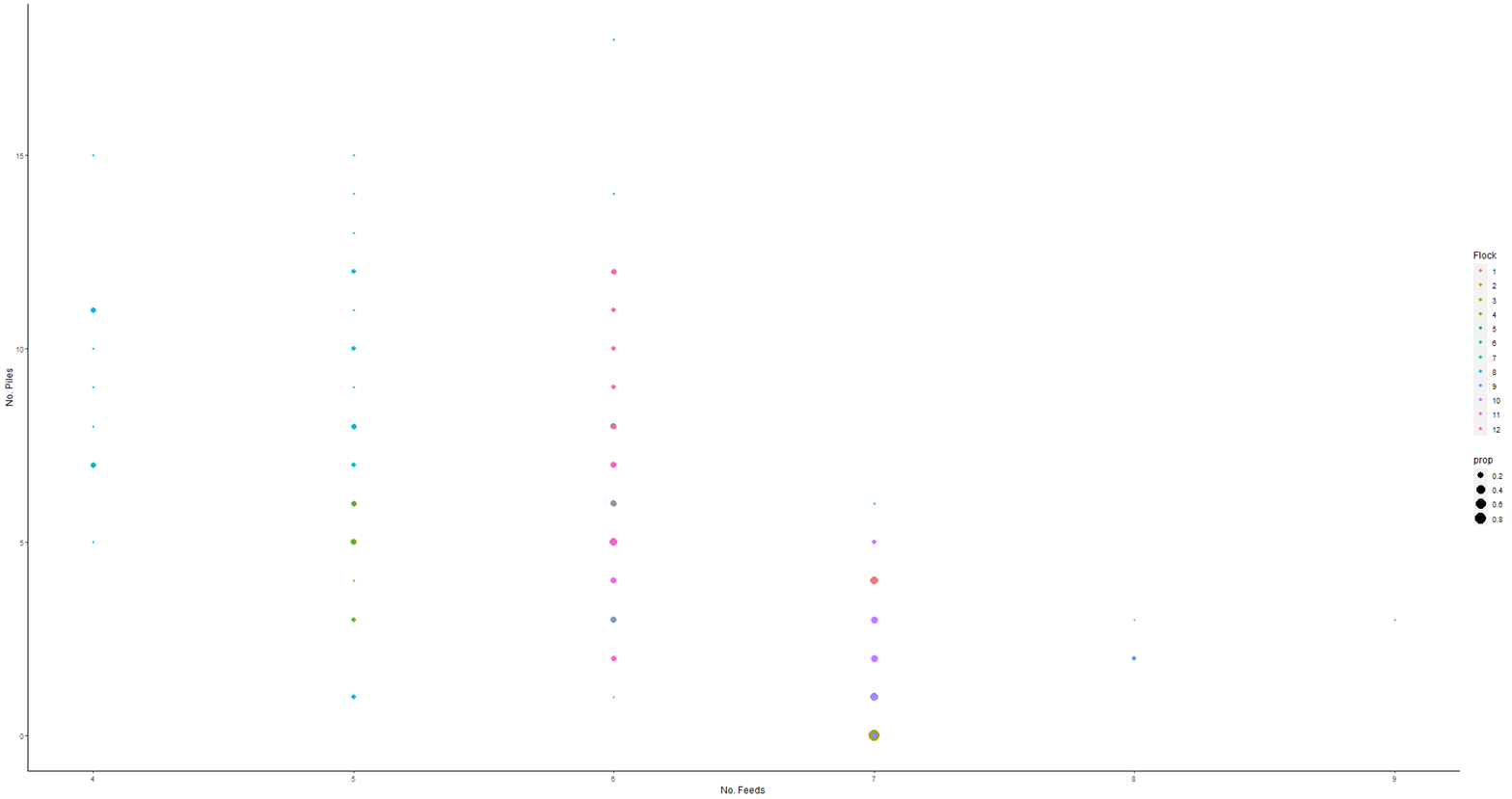


Figure S8 The effect of the number of feeds on the number of piles. The size of the point is proportional to the number of observations.


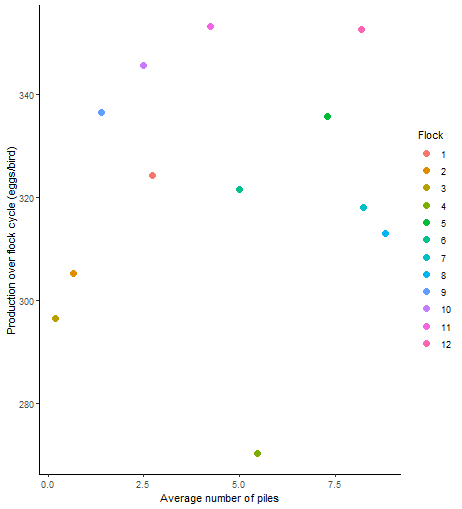


Figure S9 The effect of a flock’s propensity to pile on its lifetime production


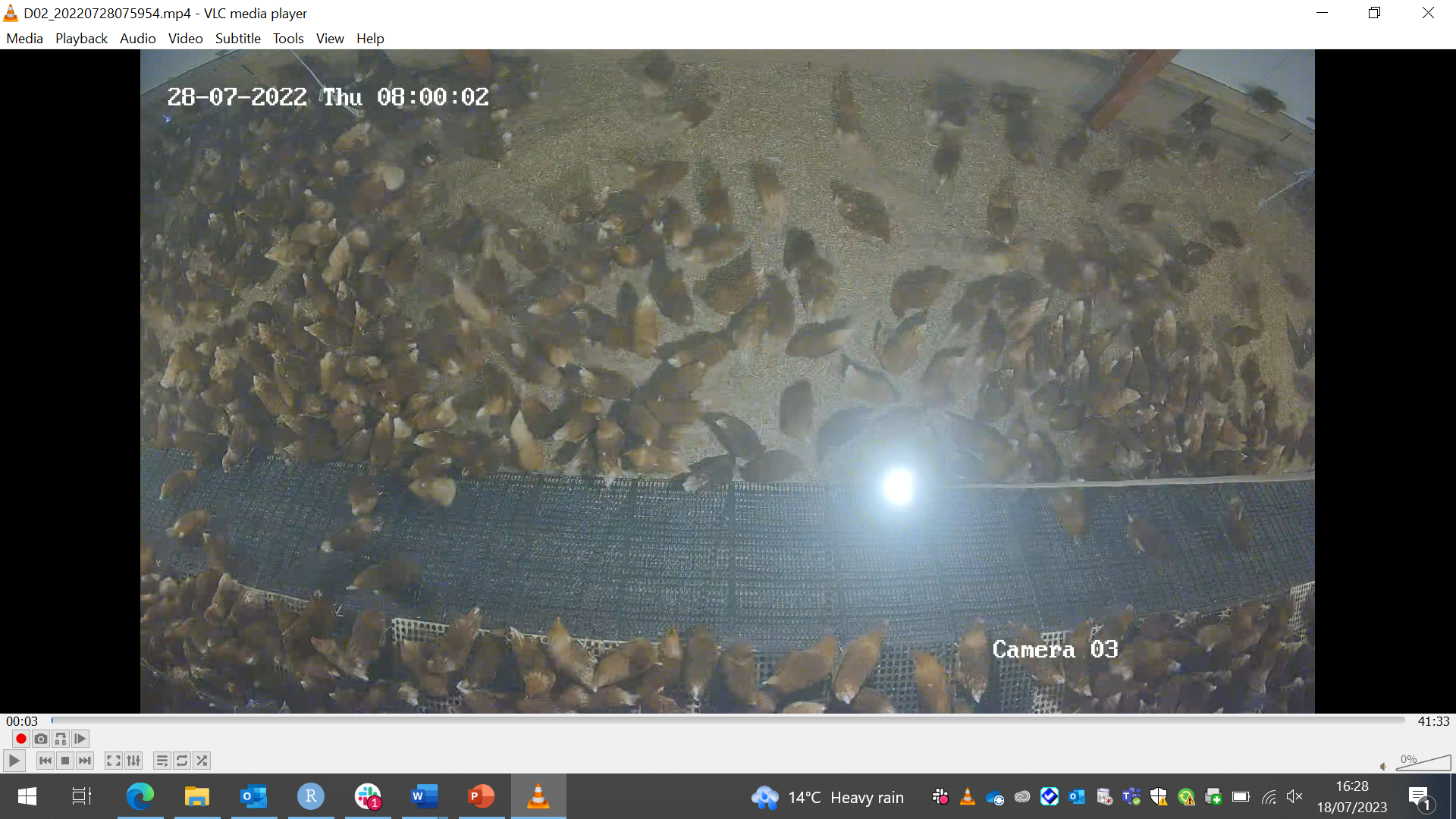


Figure S10 Screenshot from Flock 4


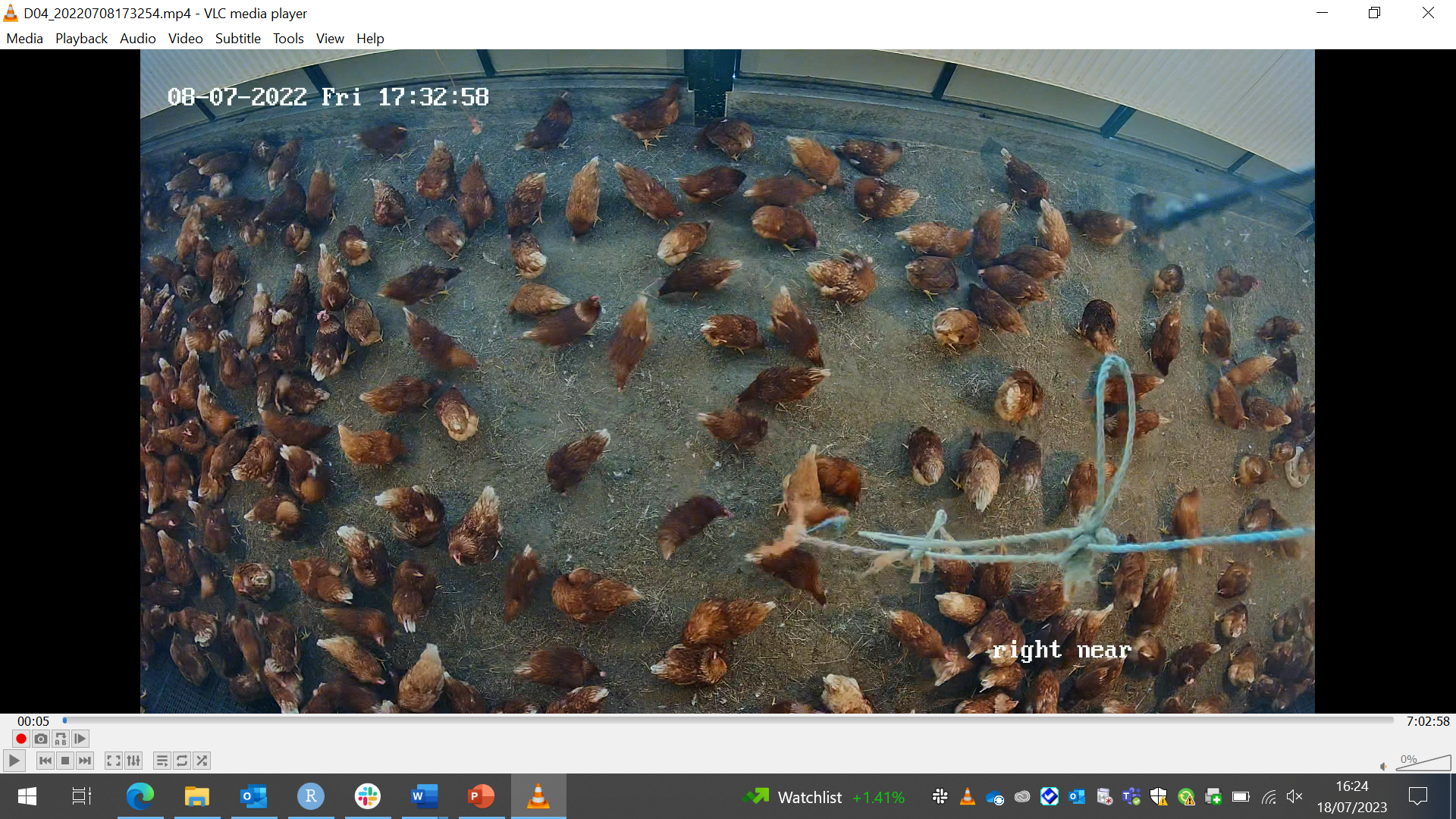


Figure S11 Screenshot from Flock 5


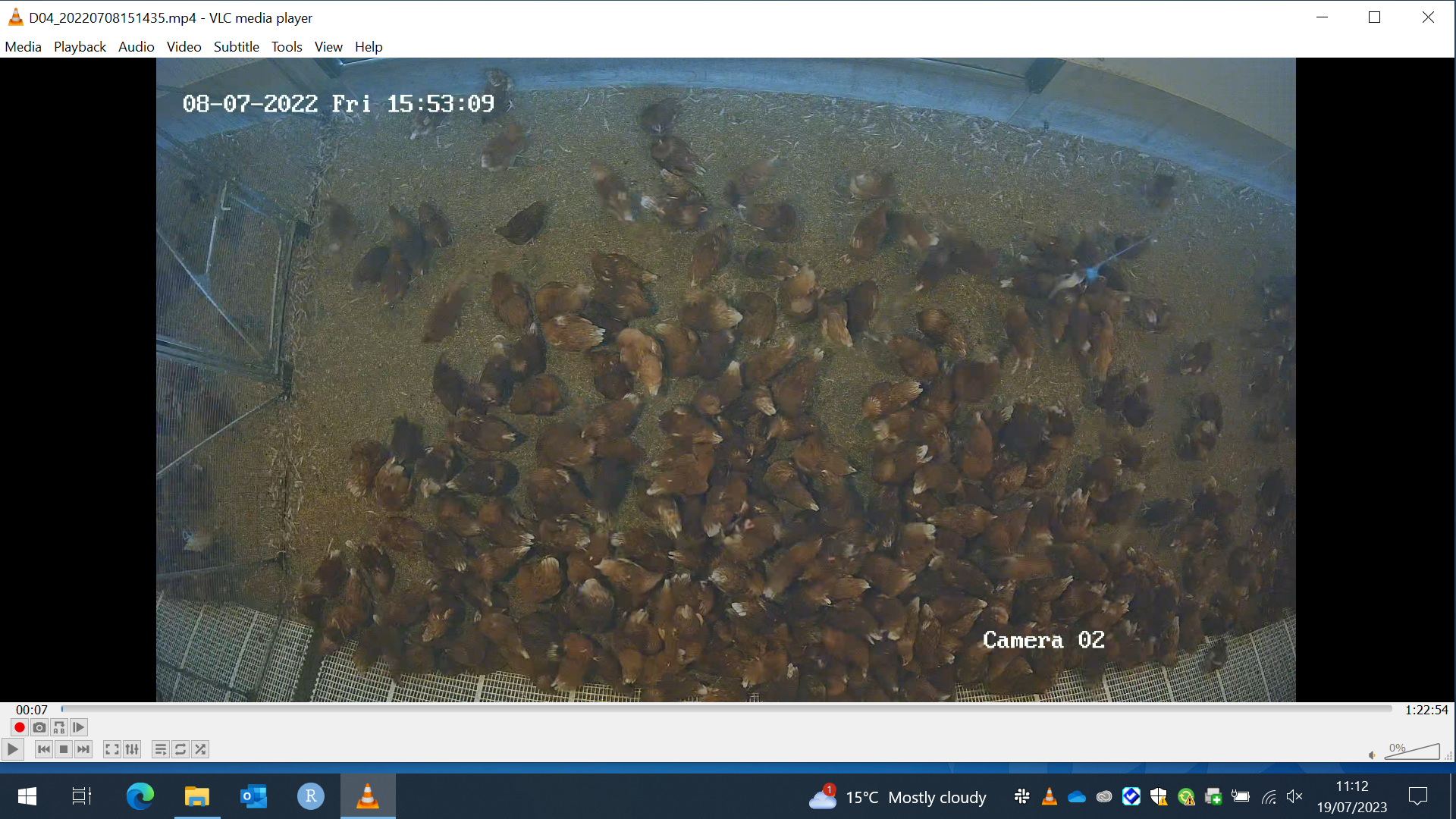


Figure S12 Screenshot from Flock 7
